# Anticoagulantes Orais Diretos versus Aspirina para Prevenção Secundária de Acidente Vascular Cerebral em Pacientes com Acidente Vascular Cerebral Embólico de Fonte Indeterminada: Revisão Sistemática e Metanálise Atualizada de Ensaios Clínicos Randomizados

**DOI:** 10.36660/abc.20240586

**Published:** 2025-06-27

**Authors:** Juan Armando Talavera, Larissa Teixeira, Thomas Costa Alexandre, Denilsa Navalha, Tathiane Brum Gibicoski, Nicole Fernandez, Jeffrey Healey, Luciana Armaganijan, Guilherme Dagostin de Carvalho

**Affiliations:** 1 Mount Sinai Medical Center Department of Internal Medicine Miami EUA Mount Sinai Medical Center, Department of Internal Medicine, Miami – EUA; 2 Universidade Federal de Campina Grande Campina Grande PB Brasil Universidade Federal de Campina Grande, Campina Grande, PB – Brasil; 3 Department of Medicine University of Colorado School of Medicine Aurora Colorado EUA Department of Medicine, University of Colorado School of Medicine, Aurora, Colorado – EUA; 4 University of Nebraska Department of Internal Medicine Omaha Nebraska EUA University of Nebraska Medical Center Department of Internal Medicine, Omaha, Nebraska – EUA; 5 Universidade Federal de Ciências da Saúde de Porto Alegre Porto Alegre RS Brasil Universidade Federal de Ciências da Saúde de Porto Alegre, Porto Alegre, RS – Brasil; 6 Catholic University of Bolivia San Pablo Faculty of Medicine Santa Cruz Bolívia Catholic University of Bolivia San Pablo Faculty of Medicine, Santa Cruz – Bolívia; 7 Population Health Research Institute McMaster University Hamilton Ontario Canadá Population Health Research Institute, McMaster University, Hamilton, Hamilton, Ontario – Canadá; 8 Departamento de Arritmias Cardíacas e Eletrofisiologia Instituto Dante Pazzanese de Cardiologia São Paulo SP Brasil Departamento de Arritmias Cardíacas e Eletrofisiologia, Instituto Dante Pazzanese de Cardiologia, São Paulo, SP – Brasil

**Keywords:** Anticoagulantes, Inibidores da Agregação Plaquetária, AVC Embólico

## Abstract

O acidente vascular cerebral (AVC) Embólico de Fonte Indeterminada (ESUS, do inglês embolic stroke of undetermined source) corresponde a cerca de 20% dos AVCs isquêmicos. O tratamento ideal para a prevenção secundária do ESUS ainda não está claro. Realizar uma revisão sistemática e metanálise de ensaios clínicos randomizados (ECRs) comparando a segurança e a eficácia dos anticoagulantes orais diretos (DOACs) versus aspirina em pacientes com ESUS. Foi realizada uma busca sistemática nas bases de dados PubMed, Embase, Cochrane e Web of Science para identificar ECRs elegíveis até março de 2024. O desfecho primário foi a recorrência de AVC, e os desfechos de segurança incluíram sangramento maior e sangramento clinicamente relevante não maior (CRNMB, clinically relevant non-major bleeding). Foram calculadas razões de chance (HRs) e intervalos de confiança (ICs) de 95% para a análise. Foram incluídos quatro RCTs, envolvendo 13.970 pacientes, dos quais metade foi randomizada para o grupo de DOACs. Durante um acompanhamento médio de 16 meses, os DOACs não reduziram significativamente a recorrência de AVC (HR: 0,95; IC 95%: 0,81-1,09; p=0,44), AVC isquêmico (HR: 0,91; IC 95%: 0,79-1,06; p=0,23), mortalidade por todas as causas (HR: 1,11; IC 95%: 0,87-1,42; p=0,40) ou sangramento maior (HR: 1,56; IC 95%: 0,85%-2,86; p=0,15) em comparação à aspirina. No entanto, os DOACs foram associados a um risco significativamente maior de CRNMB (HR: 1,54; IC 95%: 1,23-1,92; p=0,0002). A análise de subgrupos não revelou diferenças significativas na recorrência de AVC entre pacientes com escores CHA_2_-DS_2_-VASc baixos ou altos. Os DOACs não demonstraram eficácia superior à aspirina na prevenção da recorrência de AVC em pacientes com ESUS e foram associados a um aumento do risco de CRNMB.

## Graphical abstract

**Figura Central:**
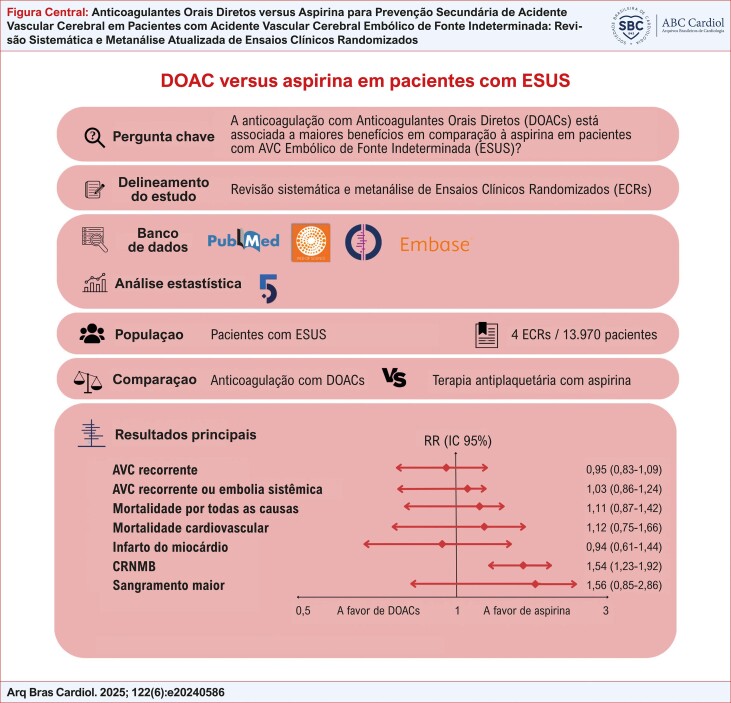
: Anticoagulantes Orais Diretos versus Aspirina para Prevenção Secundária de Acidente Vascular Cerebral em Pacientes com Acidente Vascular Cerebral Embólico de Fonte Indeterminada: Revisão Sistemática e Metanálise Atualizada de Ensaios Clínicos Randomizados

CRNMB: sangramento clinicamente relevante não maior; IC: intervalo de confiança; RR: razão de risco.

## Introdução

Os acidentes vasculares cerebrais (AVCs) isquêmicos representam aproximadamente 80% de todos os AVCs. A maioria surge da aterosclerose ou de embolia cardíaca, enquanto um terço tem uma causa incerta, denominada criptogênica.^1 - 3^ Dentro dessa categoria, o AVC Embólico de Fonte Indeterminada (ESUS, do inglês *Embolic Stroke of Undetermined Source* ) representa aproximadamente 20% dos AVCs isquêmicos que não são nem lacunares nem associados à estenose arterial proximal ou a uma fonte cardioembólica conhecida, como fibrilação atrial (FA) ou trombo ventricular esquerdo.^4^

A eficácia conhecida dos anticoagulantes orais diretos (DOACs, *direct oral anticoagulants* ) na prevenção de AVC embólico em pacientes com FA^5 , 6^ levou à hipótese de que os anticoagulantes seriam mais eficazes do que a terapia com aspirina para prevenir recorrências de AVC em pacientes com ESUS recente.^4 , 7^ No entanto, ensaios clínicos randomizados (ECRs) anteriores demonstraram que essa classe não é superior à aspirina na prevenção de eventos isquêmicos recorrentes em pacientes com ESUS.^8 - 12^

Em nosso conhecimento, existe apenas uma metanálise que avaliou essa comparação, com poder possivelmente limitado devido à inclusão de apenas dois ECRs. Nesse contexto, buscamos realizar uma revisão sistemática e metanálise atualizada de RCTs, incluindo os estudos mais recentes e disponíveis. Nosso objetivo foi comparar a eficácia e segurança da terapia anticoagulante com DOACs *versus* aspirina na prevenção de AVCs secundários em pacientes adultos com ESUS.

## Métodos

Esta revisão sistemática e metanálise seguiu as diretrizes estabelecidas no *Preferred Reporting Items for Systematic Reviews and Meta-analyses* (PRISMA) e no Manual Cochrane de Revisões Sistemáticas de Intervenções.^13 , 14^ O protocolo do estudo foi registrado prospectivamente no *International Prospective Register for Systematic Reviews* (PROSPERO) sob o número CRD42024520640. Como este estudo envolveu a análise de dados previamente publicados, não foi necessária aprovação do comitê de ética institucional nem consentimento informado dos pacientes para participação no estudo.

### Estratégia de pesquisa e extração de dados

Dois autores (A.T. e T.G.) realizaram independentemente uma busca sistemática nas bases de dados PubMed, Embase, Cochrane e Web of Science desde sua criação até março de 2024. Além disso, realizamos uma abordagem “bola de neve” (“ *backward snowballing* ”), para buscar outros estudos elegíveis a partir das referências de estudos incluídos e publicações anteriores, incluindo metanálises.

A estratégia de busca completa está disponível no Material Suplementar . Nenhum filtro ou restrição de idioma foi aplicado à pesquisa. Dois autores (A.T. e D.N.) conduziram a extração de dados seguindo critérios predefinidos e avaliação de qualidade. Características do estudo, dados demográficos iniciais, características clínicas, terapia antiplaquetária utilizada, tamanho da amostra, desfechos do estudo e o acompanhamento mais longo disponível foram extraídos diretamente dos artigos publicados informações. Divergências foram resolvidas por consenso e discussão com o autor sênior (G.D.).

### Critérios de eligibilidade

Foram incluídos os estudos que atenderam aos seguintes critérios: (1) ECR publicados em periódicos revisados por pares, (2) comparando anticoagulação oral com terapia antiplaquetária, (3) incluindo pacientes com AVC criptogênico ou ESUS, e (4) relatando pelo menos um desfecho de interesse. Não excluímos estudos com base no tamanho da amostra ou na duração do acompanhamento. Excluímos estudos que (1) não incluíam a população de interesse, (2) incluíam pacientes com Forame Oval Patente (FOP) no grupo de intervenção, sem um subgrupo de pacientes que apresentassem somente AVC criptogênico ou ESUS; (3) não relatavam nenhum dos desfechos de interesse, e (4) editoriais, levantamentos de bancos de dados nacionais, cartas ou resumos de congressos.

### Avaliação de qualidade e risco de viés

Dois autores (L.T. e T.C.) realizaram independentemente a avaliação da qualidade dos ECRs utilizando a ferramenta da Colaboração Cochrane para avaliação do risco de viés em estudos randomizados (RoB-2). Nessa abordagem, os estudos são classificados como alto, baixo ou risco incerto de viés em cinco domínios – seleção, desempenho, detecção, atrito e viés de relato.^13^ As divergências foram resolvidas por consenso. Pequenos efeitos potenciais do estudo (viés de publicação) foram avaliados por meio da análise de gráficos de funil, examinando a distribuição gráfica dos estudos com pesos semelhantes em relação aos seus erros padrão.

### Desfechos

O desfecho primário de interesse foi (1) recorrência de AVC. Os desfechos secundários incluíram: (2) o composto de recorrência de AVC ou embolia sistêmica; (3) AVC isquêmico; (4) AVC hemorrágico; (5) mortalidade por todas as causas; (6) mortalidade cardiovascular; (7) Infarto do Miocárdio (IM); (8) sangramento maior; (9) sangramento clinicamente relevante não maior (CRNMB, *clinically relevant non-major bleeding* ); (10) AVC incapacitante.

As definições dos desfechos individuais estão detalhadas na Tabela Suplementar 1 . Também realizamos uma análise de subgrupos para o desfecho de recorrência de AVC em pacientes com baixo escore CHA_2_DS_2_-VASc (definido como ≤4) e em pacientes com alto escore CHA_2_DS_2_-VASc (definido como ≥5).

### Análise estatística

Calculamos as razões de chance (HR) com intervalos de confiança (IC) de 95% para os principais desfechos binários. Para as análises de subgrupos, calculamos as razões de risco (RR) com IC de 95% para desfechos binários. Todos os desfechos foram avaliados com base na abordagem intenção de tratar, conforme relatado no ECR original. A heterogeneidade foi avaliada utilizando o teste Q de Cochrane e a estatística I^2^, com significância estabelecida em p > 0,10 e valores de I^2^ > 25%. Os modelos de efeito aleatório de Der Simonian e Laird foram aplicados para todos os desfechos com heterogeneidade significativa; caso contrário, utilizamos o modelo de efeitos fixos. As análises estatísticas foram conduzidas utilizando o software Review Manager, versão 5.4.

Além disso, realizamos uma análise de sensibilidade para avaliar o impacto de estudos individuais, removendo sequencialmente cada ECR e reanalisando os dados restantes (análise *leave-one-out* ). A dominância do estudo foi determinada quando a remoção de um estudo alterou a significância dos valores de p do tamanho do efeito agrupado, seja de significativo para não significativo ou vice-versa.

## Resultados

### Seleção e características dos estudos

**Figura 1:**
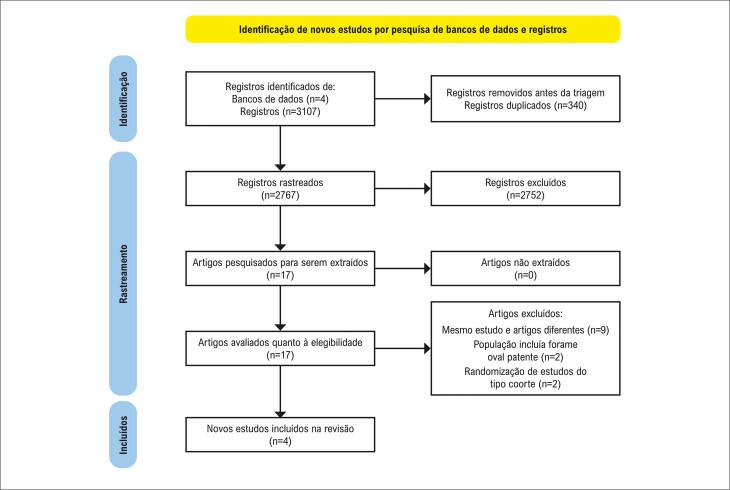
– Fluxograma PRISMA do rastreamento e seleção de estudos para a revisão sistemática e metanálise.

Conforme ilustrado na Figura 1 , nossa busca inicial resultou em 3107 registros. Após a remoção de registros duplicados e a triagem de títulos e resumos, 17 estudos continuaram elegíveis para revisão completa do texto. Desses, quatro ECRs foram incluídos.

Vale destacar que, embora tenha sido realizada uma busca ampla sobre anticoagulação *versus* terapias antiplaquetárias, todos os estudos incluídos compararam exclusivamente DOACs versus aspirina. Portanto, o presente estudo não explorou outras formas de anticoagulação ou terapias antiplaquetárias. A Figura Central resume os principais achados deste artigo.

Um total de 13 970 pacientes foi incluído, dos quais 6989 (50%) foram randomizados para o grupo de anticoagulação. O tempo médio de acompanhamento foi de 16 meses, e 39% dos pacientes eram do sexo feminino. As características do estudo e dos participantes estão detalhadas na Tabela 1 .

**Tabela 1:** – Características basais dos estudos incluídos

	NAVIGATE ESUS^11^	RE-SPECT ESUS^10^	ATTICUS^9^	ARCADIA^8^
Ano de publicação	2018	2019	2023	2024
População, n (DOAC/ aspirina)	7213 (3609/3604)	5390 (2695/2695)	352 (178/174)	1015 (507/508)
Idade, média (DP) (DOAC/ aspirina)	66,9 (9,8)/66,9 (9,8)	64,2 (11,4)/63,9 (11,4)	68,6 (10,5)/68,3 (48,9)	67,8 (10,8)/68,2 (11,0)
Sexo feminino, n (%) (DOAC/aspirina)	1377 (38,15)/ 1400 (38,84)	1001(37,1)/ 986 (36,6)	86 (48,3)/ 85 (49,9)	272 (53,7)/ 279 (54,9)
IMC (DP) Kg/m^2^ (DOAC/ aspirina)	27,1 (4,9)/27,3 (5,1)	27,2 (5)/27,3 (5)	27,7 (5,2)/27,7 (4,9)	N/A
**Comorbidades**				
Hipertensão, n (%) (DOAC/aspirina)	2782 (77)/ 2803 (78)	1996 (74,1)/ 1985 (73,7)	153 (86)/ 150 (86,2)	396 (78,1)/ 388 (76,4)
Diabetes, n (%) (DOAC/aspirina)	889 (25)/ 917 (25)	485 (21,7)/ 639 (23,7)	52 (29,2)/ 48 (27,6)	156 (30,8)/ 159 (31,3)
AVC ou AIT prévio, n (%) (DOAC/ aspirina)	620 (17,5)/643 (18)	475 (17,6)/500 (18,6)	24(13,5)/30 (17,2)	97 (19,1)100 (19,7)
Doença arterial coronariana ou isquêmica, n (%) (DOAC/ aspirina)	N/A	301 (11,2)/276 (10,2)	12 (6,7)/17 (9,8)	58 (11,4)/46 (9,1)
Insuficiência cardíaca, n (%) (DOAC/ aspirina)	N/A	117 (4,3)/ 124 (4,6)	3 (1,7)/ 4 (2,3)	36 (7,1)/35 (6,9)
Tabagismo prévio* ou atual, n (%) (DOAC/ aspirina)	756 (21)/ 728 (20)	458 (17)/ 433 (16,1)	27 (15,2)/ 26 (14,9)	230 (45,4) /200 (39,4)*
Doença arterial periférica, n (%) (DOAC/ aspirina)	N/A	N/A	7 (3,9)/9 (5,2)	12 (2,4)/ 7 (1,4)
FOP, n (%) (DOAC/ aspirina)	NA	319 (11.8)/361 (13.4)	44 (26.3)/24 (14.5)	N/A
Escore CHA_2_DS_2_-VASc médio (DP) (DOAC/ aspirina)	N/A	N/A	5 (4–6)/5 (4-5)	4.7 (1.3)/4.7 (1.3)
Escore de AVC NIH, mediano (IIQ) (DOAC/ aspirina)	1 (0–2)/1 (0-2)	1 (0–2)/1 (0-2)	1 (0–3)1 (0-3)	1 (0-3)/1 (0-3)
Dias entre AVC índice à randomização, mediana (IIQ) (DOAC/ aspirina)	38,0 (15,0–89,0)/ 36 (14,0-86,5)	46,0 (21,0–82,0)/ 43,0 (20,0-78)	8 (6–12)/ 8 (6-12)	48 (21-96)/ 53 (23-100)

AVC: acidente vascular cerebral; AIT: acidente isquêmico transitório; DP: desvio padrão; DOAC: anticoagulantes orais diretos; Forame Oval Patente (FOP); IIQ: intervalo interquartil; NIH: National Institutes of Health.

### Desfechos

#### Desfechos relacionados ao AVC

**Figura 2:**
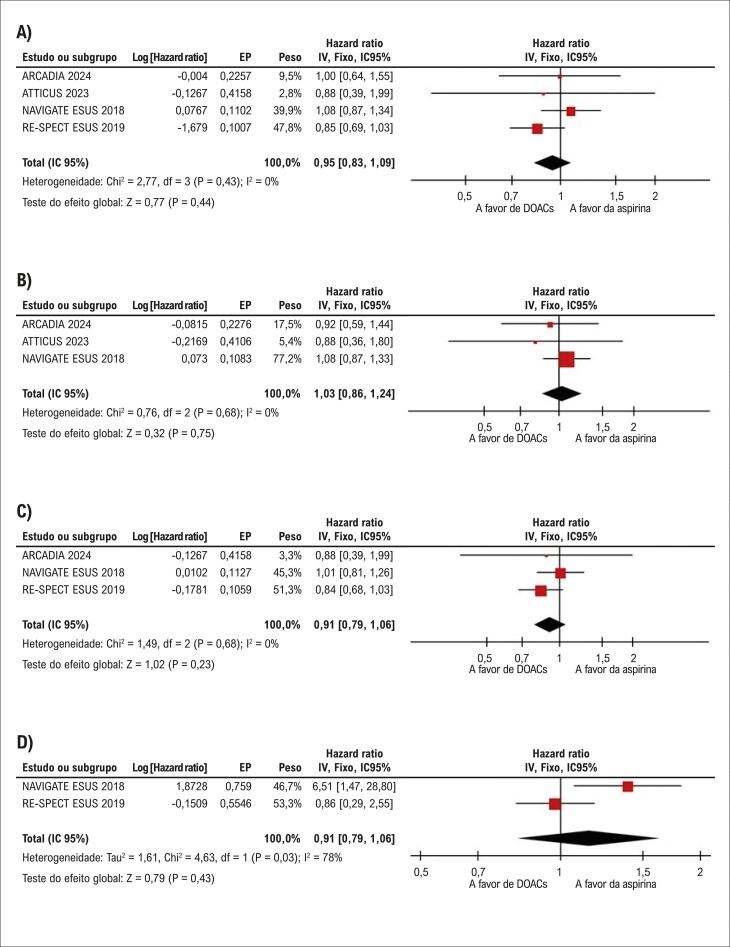
– Não houve diferenças significativas entre anticoagulantes orais diretos (DOACs) e terapia com aspirina em pacientes com acidente vascular cerebral (AVC) embólico de fonte indeterminada (ESUS) quanto à (A) recorrência de AVC, (B) desfecho composto de recorrência de AVC ou embolia sistêmica, (C) AVC isquêmico, (D) e AVC hemorrágico.

Comparados à aspirina, os DOACs não reduziram a incidência de recorrência de AVC (HR: 0,95; IC 95%: 0,83-1,09; p=0,44; I^2^=0%; Figura 2A ), do composto de recorrência de AVC ou embolia sistêmica (HR: 1,03; IC 95%: 0,86-1,24; p=0,75; I^2^=0%; Figura 2B ), e nem da incidência de AVC isquêmico recorrente (HR: 0,91; IC 95%: 0,79-1,06; p=0,23; I^2^=0%; Figura 2C ) ou AVC hemorrágico (HR: 2,21; IC 95%: 0,31-16,02; p=0,43; I^2^=78%; Figura 2D ). O AVC incapacitante foi relatado apenas por dois estudos, sem diferenças significativas entre os grupos (HR: 0,91; IC 95%: 0,39-2,16; p=0,84; I^2^=84%; Figura Suplementar 1 ).

#### Desfechos de mortalidade

**Figura 3:**
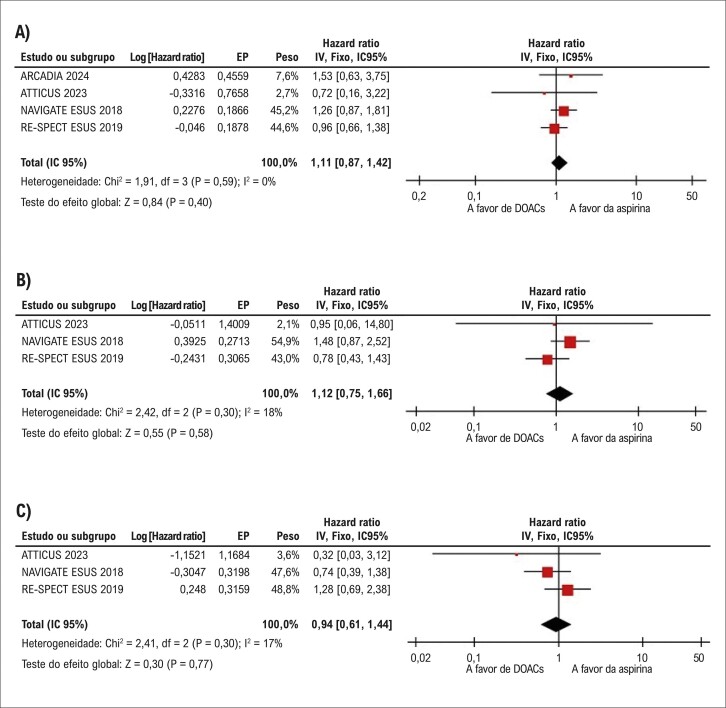
– Não houve diferenças significativas entre anticoagulantes orais diretos (DOACs) e terapia com aspirina em pacientes com acidente vascular cerebral (AVC) embólico de fonte indeterminada (ESUS) quanto à (A) mortalidade por todas as causas; (B) mortalidade cardiovascular; e (C) infarto do miocárdio.

Não houve diferenças significativas entre os grupos na incidência de mortalidade por todas as causas (HR: 1,11; IC 95%: 0,87-1,42; p=0,40; I^2^=0%; Figura 3A ) e mortalidade cardiovascular (HR: 1,12; IC 95%: 0,75-1,66; p=0,58; I^2^=18%; Figura 3B ).

#### Infarto do miocárdio

O IM foi relatado em três estudos. Não foram observadas diferenças significativas entre os grupos (HR: 0,94; IC 95%: 0,61-1,44; p=0,77; I^2^=17%; Figura 3C .

#### Desfechos de sangramento

**Figura 4:**
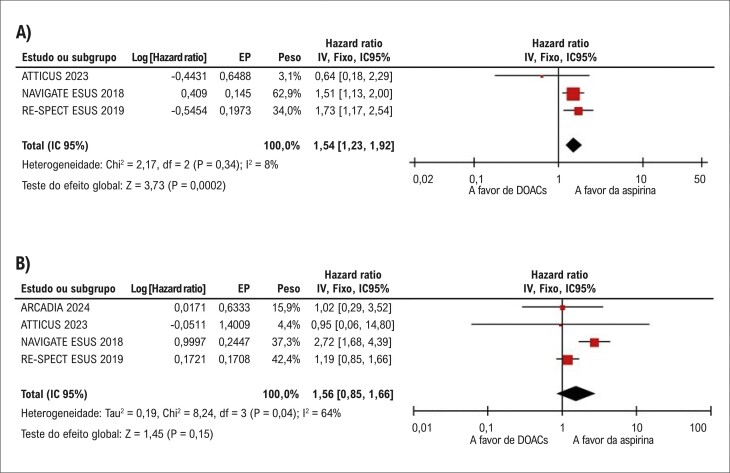
– Em comparação à terapia antiplaquetária, os anticoagulantes orais diretos (DOACs) aumentaram significativamente a ocorrência de (A) sangramento clinicamente relevante não maior; não houve diferenças significativas entre os grupos quanto a (B) sangramento maior.

Não houve diferenças significativas entre os grupos na incidência de sangramento maior (HR: 1,56; IC 95%: 0,85-2,86; p=0,15; I^2^=64%; Figura 4A ). No entanto, comparados à aspirina, os DOACs aumentaram significativamente a incidência de CRNMB (HR: 1,54; IC 95%: 1,23-1,92; p=0,0002; I^2^=8%; Figura 4B ).

#### Análises de subgrupos

Não houve diferenças significativas na recorrência de AVC entre DOACs e terapia com aspirina tanto no subgrupo de pacientes com baixo escore CHA_2_DS_2_-VASc (definido como ≤4) (RR: 0,87; IC 95%: 0,67-1,13; p=0,28; I^2^=0%; Figura Suplementar 2A ) como no de pacientes com escore CHA_2_DS_2_-VASc elevado (definido como ≥5) (RR: 0,83; IC 95%: 0,65-1,07; p=0,16; I^2^=0%; Figura Suplementar 2B ).

#### Avaliação de qualidade e análise de sensibilidade

Conforme ilustrado na Figura Suplementar 3 , todos os estudos incluídos foram considerados de baixo risco de viés.^8 - 11^ A análise de sensibilidade *leave-one-out* para o desfecho de CRNMB apresentou resultados consistentes, sem evidência de dominância de estudo ( Figura Suplementar 4 ). A análise do gráfico de funil do desfecho de CRNMB não demonstrou efeito potencial de estudo pequeno, exibindo uma distribuição gráfica simétrica dos estudos com pesos similares em relação aos seus erros padrão ( Figura Suplementar 5 ).

## Discussão

Nesta metanálise de quatro ECRs, DOACs foram comparados à aspirina em 13970 pacientes com histórico de ESUS. Os principais achados foram: não observamos diferenças estatisticamente significativas entre os dois grupos quanto à recorrência de AVC, aos desfechos compostos de recorrência de AVC ou embolia sistêmica, AVC isquêmico, AVC hemorrágico, mortalidade por todas as causas, mortalidade cardiovascular, infarto do miocárdio, sangramento maior ou AVC incapacitante. No entanto, os pacientes tratados com DOACs apresentaram uma maior incidência de CRNMB em comparação a aqueles tratados com aspirina.^12^

A premissa de que a anticoagulação oral é mais eficaz do que a terapia antiplaquetária na prevenção de AVCs relacionados à FA está bem estabelecido.^15^ Dado o alto índice de detecção de FA, que pode chegar a 16% em pacientes com AVC criptogênico durante três a 12 meses de monitoramento cardíaco contínuo, o racional para explorar a anticoagulação nesse contexto é inquestionável.^16 , 17^

Os primeiros estudos comparando DOACs com aspirina não demonstraram uma superioridade clara da anticoagulação em pacientes com ESUS.^10 , 11^ Essas descobertas levaram a novos estudos para investigar subgrupos de pacientes com ESUS, incluindo aqueles com maior risco de FA e cardioembolismo, como indivíduos com cardiopatia atrial.^8 , 9^ Em um estudo importante,^9^ essa abordagem deu origem ao conceito de “ESUS enriquecido”, distinguindo-se da população de “ESUS não selecionado” que participou de estudos anteriores. Apesar desses esforços para estratificar os pacientes com ESUS de forma mais precisa, estudos subsequentes refletiram os achados iniciais de que a anticoagulação não era superior à terapia antiplaquetária.^8 , 9^

Um subestudo do ensaio ARTESIA demonstrou que o escore CHA_2_DS_2_-VASc basal ajuda a identificar e orientar a anticoagulação oral em pacientes com FA subclínica.^18^ Para um escore CHA_2_DS_2_-VASc > 4, os benefícios do tratamento com apixabana na prevenção de AVC embólico foram maiores do que os riscos. Não encontramos diferenças significativas entre os grupos em relação à recorrência de AVC no subgrupo de pacientes com escores CHA_2_DS_2_-VASc baixos e altos.^19^

Em uma metanálise publicada anteriormente de dois ECRs, a terapia de anticoagulação não reduziu a recorrência de AVC nem aumentou os eventos hemorrágicos, incluindo sangramento maior e eventos CRNMB, quando comparada à terapia antiplaquetária.^12^ Em alinhamento com esses resultados, não encontramos diferença significativa na eficácia dos DOACs para o manejo do ESUS. No entanto, nossos achados sugerem que essa modalidade terapêutica pode aumentar o risco de CRNMB para essa população.

Considerando esses achados coletivos, nossa metanálise destaca a ausência de benefício evidente da anticoagulação em pacientes com ESUS, independentemente dos perfis de risco. Apesar da justificativa convincente para a anticoagulação com base nas taxas de detecção de FA e nas considerações fisiopatológicas teóricas, os dados disponíveis não demonstraram que a terapia com DOACs seja superior à aspirina na redução da recorrência de AVCs em pacientes com ESUS.

Em nosso conhecimento, esta é uma das maiores metanálises restritas a ECRs comparando DOACs à aspirina em pacientes com ESUS. Ao reunir um conjunto de dados mais amplo, nossos resultados aumentam o poder estatístico, oferecendo uma perspectiva valiosa sobre o debate contínuo em torno do manejo e das estratégias de prevenção do AVC. Além disso, nosso estudo apresenta pontos fortes consideráveis, pois a avaliação rigorosa determinou que todos os ensaios incluídos tinham baixo risco de viés. A maioria desses estudos tinha um delineamento duplo-cego,^8 , 10 , 11^ juntamente com adjudicadores cegos de desfechos,^8 - 11^ o que reduz significativamente o potencial de viés.

Embora forneça *insights* valiosos, nosso estudo apresenta limitações. Primeiro, observamos variações entre os estudos nas definições de comorbidades, nos critérios de seleção dos ensaios e na duração do acompanhamento. Segundo, as estimativas de duração do acompanhamento foram baseadas em médias relatadas, o que pode ter negligenciado benefícios significativos do tratamento sugeridos por divergências tardias nas curvas de Kaplan–Meier, como observado no estudo RE-SPECT ESUS. Além disso, identificamos uma heterogeneidade moderada a alta no desfecho de sangramento maior, o que pode refletir as variações mencionadas entre os estudos. Para mitigar essa influência na análise agrupada, utilizamos um modelo de efeitos aleatórios. Por fim, embora tenha sido realizada uma ampla busca por estudos comparando terapia anticoagulante com terapia antiplaquetária, todos os estudos incluídos compararam exclusivamente DOACs com aspirina. Consequentemente, nossa análise pós-busca foi especificamente direcionada para DOACs *versus* aspirina, excluindo comparações com outras formas de anticoagulação ou tratamentos antiplaquetários. Assim, os desfechos de nossa metanálise podem não ser amplamente aplicáveis a outros regimes anticoagulantes ou antiplaquetários.

Marcadores de cardiopatia atrial, como o índice de volume do átrio esquerdo e os níveis de NT-pro-BNP, mostram potencial para estratificar os desfechos de ESUS. No entanto, devido à ausência de dados individuais, esta metanálise não pôde explorar tais subgrupos. Em relação aos estudos individuais, o ensaio ARCADIA não encontrou diferença significativa na recorrência de AVC entre apixabana e aspirina em pacientes com NT-pro-BNP >250 pg/mL ou um índice de diâmetro do átrio esquerdo >1,8 cm^2^.^8^No entanto, outros ensaios, incluindo ATTICUS, NAVIGATE ESUS e RE-SPECT ESUS, não relataram análises de subgrupos para esses marcadores.^9 - 11^Pesquisas futuras que integrem marcadores de cardiopatia atrial podem identificar melhor os subgrupos que se beneficiam da terapia anticoagulante.

Portanto, ao selecionar estratégias de tratamento para pacientes com ESUS, os profissionais de saúde devem pesar cuidadosamente os benefícios potenciais dos DOACs contra os riscos associados de sangramento. De modo geral, nossos resultados indicam que a terapia antiplaquetária com aspirina pode ser uma opção mais segura para pacientes com ESUS. No entanto, é importante mencionar que seu uso ainda apresenta um risco substancial de recorrência de AVC nessa população, o que ressalta a importância de explorar outras abordagens. Assim, pesquisas futuras são essenciais para identificar estratégias alternativas de manejo e melhorar os desfechos para pacientes com ESUS.

## Conclusão

Nesta metanálise de quatro ECRs, constatamos que o uso de DOACs não foi superior à aspirina na prevenção da recorrência de AVC em pacientes com ESUS. Além disso, houve um aumento estatisticamente significativo na ocorrência de CRNMB no grupo tratado com DOACs, em comparação com o grupo tratado com aspirina, apesar de não haver diferença significativa na incidência de sangramento maior.

## *Material suplementar

Para informação adicional, por favor, clique aqui


