# Errata

**DOI:** 10.36660/abc.20250535

**Published:** 2025-08-06

**Edição de Junho de 2025, vol. 122(6):e20240586**

No Artigo de Revisão "Anticoagulantes Orais Diretos versus Aspirina para Prevenção Secundária de Acidente Vascular Cerebral em Pacientes com Acidente Vascular Cerebral Embólico de Fonte Indeterminada: Revisão Sistemática e Metanálise Atualizada de Ensaios Clínicos Randomizados", com número de DOI: https://doi.org/10.36660/abc.20240586 , publicado no periódico Arquivos Brasileiros de Cardiologia, Arq Bras Cardiol. 2025; 122(6):e20240586, na página 1, alterar o nome do autor Thomas Costa Alexandre para: Thomaz Alexandre Costa.

