# Supplementary material for: Anticoagulantes Orais Diretos versus Aspirina para Prevenção Secundária de Acidente Vascular Cerebral em Pacientes com Acidente Vascular Cerebral Embólico de Fonte Indeterminada: Revisão Sistemática e Metanálise Atualizada de Ensaios Clínicos Randomizados
Source: Arq Bras Cardiol. 2025 Jun 27;122(6):e20240586. [Article in Portuguese] doi: 10.36660/abc.20240586 (PMC12269895; doi:10.36660/abc.20240586)
Supplement: Supplementary file 1 [file 2024-0586_AR_Supplemental_Material_ESUS_meta.pdf]

Supplemental Material

Complete Search Strategy

("Embolic Stroke of Undetermined Source" OR ESUS OR "Embolic stroke of unknown source" OR "Cryptogenic Stroke" OR "Embolic stroke") AND ("oral anticoagulation" OR dabigatran OR noac OR doac OR rivaroxaban OR apixaban OR edoxaban OR warfarin OR "vitamin K antagonist") AND ("antiplatelet therapy" OR aspirin OR "acetylsalicylic acid" OR ASA OR Clopidogrel OR Ticagrelor OR Prasugrel) AND (RCT OR "randomized controlled trial" OR random OR randomly OR randomized OR randomization OR "randomized clinical trial")

Supplemental tables

Table S1. Summary definitions of outcomes in each included trial

Trial	Outcome definition
**NAVIGATE ESUS**[1]	**Recurrent Stroke**<br>Included ischemic, hemorrhagic, or undefined stroke<br><br>**Ischemic Stroke**<br>Defined as a focal neurologic deficit of sudden onset that was due to presumed arterial occlusion persisting for more than 24 hours and without evidence of primary hemorrhage on neuroimaging; if there was a neurologic deficit lasting less than 24 hours, evidence of acute brain infarct had to be present on neuroimaging.[2]<br><br>**Hemorrhagic stroke**<br>Included symptomatic, nontraumatic intracerebral and subarachnoid hemorrhages.<br><br>**Undefined stroke**<br>Definition based on an absence of neuroimaging or autopsy features to distinguish ischemic from hemorrhagic stroke, were considered to be ischemic strokes in the analyses unless otherwise noted.<br><br>**Cardiovascular death**<br>Death due to cardiovascular causes.<br><br>**Disabling stroke**<br>Defined according to modified Rankin scale score of 4 or 5 at hospital discharge.<br><br>**Major Bleeding**

1

	Defined according to the criteria of the International Society of Thrombosis and Hemostasis (ISTH).[3]<br><br>**Clinically Relevant nonmajor bleeding**<br>Defined according to the criteria of the International Society of Thrombosis and Hemostasis (ISTH).[33]
**RE-SPECT ESUS**[4]	**Recurrent Stroke**<br>Included ischemic, hemorrhagic, or unspecified type.<br><br>**Ischemic Stroke**<br>Included undefined strokes with no neuroimaging/autopsy.<br><br>**Hemorrhagic stroke**<br>Included primary intracerebral and subarachnoid hemorrhage.<br><br>**Disabling stroke**<br>Defined by a score on the modified Rankin scale of 4 or more 3 months after a recurrent stroke.<br><br>**Major Bleeding**<br>Defined according to International Society on Thrombosis and Hemostasis (ISTH) criteria.[3]<br><br>**Clinically Relevant nonmajor bleeding**<br>Defined as nonmajor bleeding resulting in hospitalization, medical or surgical intervention, or change, interruption, or discontinuation of the trial drug.
**ATTICUS**[5]	**Recurrence stroke**<br>Defined as any new ischemic lesion on diffusion-weighted or fluid-attenuated inversion recovery brain magnetic resonance imaging (MRI) compared with baseline during 12-month follow-up.<br><br>**Major Bleeding**<br>Defined according to the International Society on Thrombosis and Hemostasis.[3]<br><br>**Clinically relevant nonmajor bleeding**<br>Defined according to the International Society on Thrombosis and Hemostasis.[3]
**ARCADIA**[6]	**Recurrent Stroke**<br>Included ischemic, hemorrhagic, or undetermined type.<br><br>**Ischemic Stroke**

	Defined as a rapid onset of a new focal neurological deficit: 1) imaging or other evidence of infarction in a part of the central nervous system consistent with symptoms OR 2) lasting >24 hours without imaging evidence of infarction, AND 3) not attributable to a non-ischemic etiology, such as intracranial hemorrhage, edema, infection, trauma, tumor, seizure, severe metabolic disease, or degenerative neurological disease<br><br>**Hemorrhagic stroke**<br>Defined as an acute extravasation of blood into the brain parenchyma, subarachnoid space, or intraventricular space, judged to be non- traumatic and not in the area of an acute or subacute ischemic infarct but associated with and identified as the predominant cause of new neurologic symptoms, including headache, or leading to death.<br><br>**Stroke of undetermined type:**<br>Defined as a rapid onset of a new focal neurological deficit: 1) lasting >24 hours AND 2) not meeting criteria for ischemic stroke or hemorrhagic stroke AND 3) not attributable to a non-vascular etiology, such as edema, infection, trauma, tumor, seizure, severe metabolic disease, or degenerative neurological disease.<br><br>**Systemic embolism**<br>Defined as a clinical history consistent with an acute loss of blood flow to a peripheral artery or arteries and supported by evidence of embolism from surgical specimens, autopsy, angiography, or other objective testing.<br><br>**Major Bleeding**<br>Defined as a clinically overt bleeding accompanied by a 2-g/dL or greater decrease in the hemoglobin level during a 24-hour period, transfusion of 2 units or more of whole blood or red blood cells, involvement of a critical non-intracranial site (intraspinal, intraocular, pericardial, intra-articular, intramuscular with compartment syndrome, or retroperitoneal), or death.[7]

ISTH: International Society of Thrombosis and Hemostasis.

Supplemental Figures

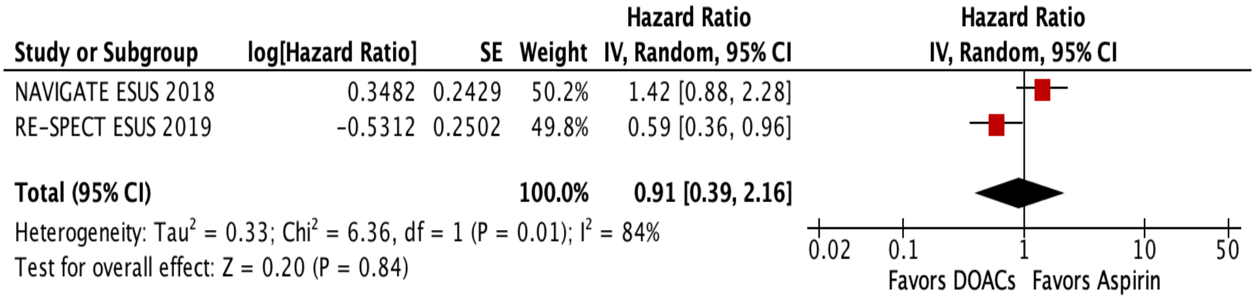

Fig. S1 There is no significant differences between groups in the incidence of disabling stroke.

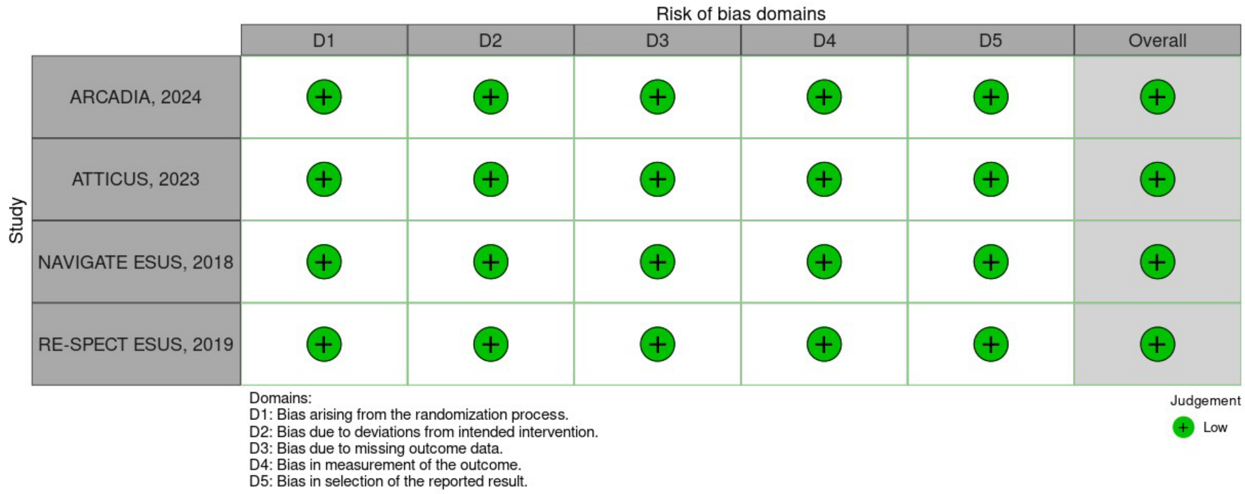

Fig. S2 Risk of bias assessment of the included randomized controlled trials according to the Cochrane's RoB-2.

REFERENCES

1. Hart RG, Sharma M, Mundl H, Kasner SE, Bangdiwala SI, Berkowitz SD, et al. Rivaroxaban for Stroke Prevention after Embolic Stroke of Undetermined Source. *N Engl J Med.* 2018;378(23):2191–201. doi:10.1056/NEJMoa1802686

2. Hicks KA, Mahaffey KW, Mehran R, Nissen SE, Wiviott SD, Dunn B, et al. 2014 ACC/AHA Key Data Elements and Definitions for Cardiovascular Endpoint Events in Clinical Trials. *J Am Coll Cardiol.* 2015;66(4):403–69. doi:10.1016/j.jacc.2014.12.018

3. Schulman S, Kearon C. Definition of major bleeding in clinical investigations of antihemostatic medicinal products in non-surgical patients. *J Thromb Haemost.* 2005;3(4):692–4. doi:10.1111/j.1538-7836.2005.01204.x

4. Diener H-C, Sacco RL, Easton JD, Granger CB, Bernstein RA, Uchiyama S, et al. Dabigatran for Prevention of Stroke after Embolic Stroke of Undetermined Source. *N Engl J Med.* 2019;380(20):1906–17. doi:10.1056/NEJMoa1813959

5. Geisler T, Poli S, Ziegler A, Norrving B, Diener H-C, Hart RG, et al. Apixaban versus Aspirin for Embolic Stroke of Undetermined Source. *NEJM Evid.* 2023;3. doi:10.1056/EVIDoa2300018

6. Kamel H, Longstreth WT, Tirschwell DL, Kronmal RA, Elkind MSV, Samuels KA, et al. Apixaban to Prevent Recurrence After Cryptogenic Stroke in Patients With Atrial Cardiopathy. *JAMA.* 2024;331(6):573. doi:10.1001/jama.2024.0092

7. Schulman S, Angerås U, Bergqvist D, Eriksson B, Lassen MR, Fisher W, et al. Definition of major bleeding in clinical investigations of antihemostatic medicinal products in surgical patients. *J Thromb Haemost.* 2010;8(1):202–4. doi:10.1111/j.1538-7836.2009.03678.x
