# Supplementary material for: Anticoagulantes Orais Diretos versus Aspirina para Prevenção Secundária de Acidente Vascular Cerebral em Pacientes com Acidente Vascular Cerebral Embólico de Fonte Indeterminada: Revisão Sistemática e Metanálise Atualizada de Ensaios Clínicos Randomizados
Source: Arq Bras Cardiol. 2025 Jun 27;122(6):e20240586. [Article in Portuguese] doi: 10.36660/abc.20240586 (PMC12269895; doi:10.36660/abc.20240586)
Supplement: Translation

# Translation: Direct Oral Anticoagulants versus Aspirin for Secondary Stroke Prevention in Patients with Embolic Stroke of Undetermined Source: An Updated Systematic Review and Meta-Analysis of Randomized Controlled Trials

**DOI:** 10.36660/abc.20240586i

**Authors:** Juan Armando Talavera, Larissa Teixeira, Thomas Costa Alexandre, Denilsa Navalha, Tathiane Brum Gibicoski, Nicole Fernandez, Jeffrey Healey, Luciana Armaganijan, Guilherme Dagostin de Carvalho

**Affiliations:** 1 USA Mount Sinai Medical Center, Department of Internal Medicine, Miami – USA; 2 Brazil Universidade Federal de Campina Grande, Campina Grande, PB – Brazil; 3 USA Department of Medicine, University of Colorado School of Medicine, Aurora, Colorado – USA; 4 USA University of Nebraska Medical Center Department of Internal Medicine, Omaha, Nebraska – USA; 5 Brazil Universidade Federal de Ciências da Saúde de Porto Alegre, Porto Alegre, RS – Brazil; 6 Bolivia Catholic University of Bolivia San Pablo Faculty of Medicine, Santa Cruz – Bolivia; 7 Canada Population Health Research Institute, McMaster University, Hamilton, Hamilton, Ontario – Canada; 8 Brazil Departamento de Arritmias Cardíacas e Eletrofisiologia, Instituto Dante Pazzanese de Cardiologia, São Paulo, SP – Brazil

**Keywords:** Anticoagulants, Platelet Aggregation Inhibitors, Embolic Stroke

## Abstract

Embolic stroke of undetermined source (ESUS) accounts for around 20% of ischemic strokes. The ideal treatment for secondary prevention in ESUS remains unclear. This study aimed to perform a systematic review and meta-analysis of randomized controlled trials (RCTs) comparing the safety and efficacy of direct oral anticoagulants (DOACs) versus aspirin in patients with ESUS. A systematic search of PubMed, Embase, Cochrane, and Web of Science databases was conducted for eligible trials until March 2024. The primary outcome was recurrent stroke, while safety outcomes included major bleeding and clinically relevant non-major bleeding (CRNMB). Hazard ratios (HRs) with 95% confidence intervals (CIs) were calculated for analysis. Four RCTs were included, involving 13,970 patients, half of whom were randomized to the DOACs group. Over a mean follow-up of 16 months, DOACs did not significantly reduce recurrent stroke (HR: 0.95; 95% CI: 0.81-1.09; p=0.44), ischemic stroke (HR: 0.91; 95% CI: 0.79-1.06; p=0.23), all-cause mortality (HR: 1.11; 95% CI: 0.87-1.42; p=0.40), or major bleeding (HR: 1.56; 95% CI: 0.85%-2.86; p=0.15) compared to aspirin. However, DOACs were associated with a significantly higher risk of CRNMB (HR: 1.54; 95% CI: 1.23-1.92; p=0.0002). Subgroup analysis revealed no significant differences in stroke recurrence among patients with low or high CHA_2_-DS_2_-VASc scores. DOACs did not demonstrate superior efficacy over aspirin in preventing recurrent stroke among ESUS patients and were linked to an increased risk of CRNMB.

## Introduction

Ischemic strokes account for roughly 80% of all strokes.^1^The majority arises from atherosclerosis or heart embolism, while one-third has an unclear cause, termed cryptogenic.^1 - 3^Within this category, embolic stroke of undetermined source (ESUS) represents approximately 20% of ischemic strokes that are neither lacunar nor associated with proximal arterial stenosis or a recognized cardioembolic source, such as consequent of atrial fibrillation (AF) or a left ventricular thrombus.^4^

The known efficacy of direct oral anticoagulants (DOACs) for the prevention of embolic stroke in patients with AF^5 , 6^led us to hypothesize that anticoagulants would be more effective than aspirin therapy for the prevention of recurrent stroke in patients with recent ESUS.^4 , 7^However, previous randomized clinical trials (RCTs) have demonstrated non-superiority of this class in preventing recurrent ischemic events in patients with ESUS when compared to aspirin.^8 - 12^

To the best of our knowledge, there is only one meta-analysis assessing this comparison, with potentially limited power due to the inclusion of only two RCTs. In this context, we aimed to conduct an updated systematic review and meta-analysis of RCTs, including the latest and most current studies available. Our goal was to compare the efficacy and safety of anticoagulation therapy with DOACs versus aspirin in preventing secondary strokes in adult patients with ESUS.

## Methods

This systematic review and meta-analysis followed the guidelines outlined in the Preferred Reporting Items for Systematic Reviews and Meta-analyses (PRISMA) and the Cochrane Handbook of Systematic Reviews of Interventions.^13 , 14^The study protocol was prospectively registered in the (PROSPERO) under protocol number CRD42024520640. As this study involved the analysis of previously published data, Institutional Review Board approval and informed patient consent for study participation were not required.

### Search strategy and data extraction

Two authors (A.T. and T.G.) independently performed a systematic search of PubMed, Embase, Cochrane and Web of Science databases from inception through March 2024. Additionally, we performed a “backward snowballing” searching for additional eligible studies through review of references of included studies and prior publications, including meta-analyses.

The complete search strategy is available in the Supplemental Material. No filters or language limitations were applied to our search. Two authors (A.T. and D.N.) conducted the data extraction following predefined criteria and quality assessment. Study characteristics, baseline demographics, clinical characteristics, antiplatelet therapy used, sample size, study outcomes, and the longest available follow-up were extracted directly from the published articles. Disagreements were resolved through consensus and discussion with the senior author (G.D.).

### Eligibility criteria

Studies that met the following criteria were included: (1) RCTs published in peer review journals, (2) comparing oral anticoagulation with antiplatelet therapy, (3) enrolling patients with cryptogenic stroke or ESUS, and (4) reporting at least one endpoint of interest. We did not exclude studies based on sample size or follow-up duration. We excluded studies that (1) did not include the population of interest, (2) included patients with patent foramen ovale (PFO) in the interventional group when a subgroup of patients with only cryptogenic stroke or ESUS was not available; (3) studies not reporting any of the outcomes of interest, and (4) editorials, national database surveys, letters, or conference abstracts.

### Quality assessment, risk of bias

Two authors (L.T. and T.C.) independently conducted the quality assessment of RCTs using the Cochrane Collaboration’s tool for assessing risk of bias in randomized trials (RoB-2), in which studies are graded as high, low, or unclear risk of bias in five domains – selection, performance, detection, attrition, and reporting biases.^13^Disagreements were resolved by consensus. Small potential study effects (publication bias) were evaluated by funnel plots analysis of the graphical distribution of studies with similar weights against their standard errors.

### Outcomes

The primary endpoint of interest was (1) stroke recurrence. Secondary endpoints included (2) the composite of recurrent stroke or systemic embolism; (3) ischemic stroke; (4) hemorrhagic stroke; (5) all-cause mortality; (6) cardiovascular mortality; (7) myocardial infarction (MI); (8) major bleeding; (9) clinically relevant non-major bleeding (CRNMB); and (10) disabling stroke.

The definitions for the individual outcomes are detailed in the Supplementary Table 1 . We also performed a subgroup analysis for the outcome of stroke recurrence for patients with low CHA_2_-DS_2_-VASc score (defined as ≤4) and for patients with high CHA_2_-DS_2_-VASc score (defined as ≥ 5).

### Statistical analysis

We computed hazard ratios (HR) with 95% confidence intervals (CI) for the main binary outcomes. For subgroup analyses, we computed risk ratios (RR) with 95% CI for binary outcomes. All outcomes were assessed based on intention-to-treat, as reported in the original RCT. Heterogeneity was assessed using the Cochrane Q-test and I^2^statistics, with significance set at p values > 0.10 and I^2^values > 25%. Der Simonian and Laird random-effect models were applied for all outcomes with a significant heterogeneity; otherwise we used fixed-effects model. Statistical analyses were conducted using Review Manager software, version 5.4.

Furthermore, we performed a sensitivity analysis to evaluate the impact of individual studies by sequentially removing each RCT and reanalyzing the remaining data (leave-one-out analysis). Study dominance was determined when removing a study changed the significance of the pooled effect size p-values, either from significant to non-significant or vice versa.

## Results

### Study selection and characteristics

As depicted in Figure 1 , our initial search yielded 3,107 results. After removing duplicate records and conducting title and abstract screening, 17 studies remained eligible for full-text review. Of these, four RCTs were included.

**Figure 1:** – PRISMA flowchart of study screening and selection for systematic review and meta-analysis.

Of note, although a broad search was applied for anticoagulation versus antiplatelet therapies, all included studies exclusively compared DOACs *versus* aspirin. Therefore, the present study did not explore other forms of anticoagulation or antiplatelet therapies. The Central Illustration summarizes the main findings of this paper.

A total of 13,970 patients were included, of whom 6,989 (50%) were randomized to the anticoagulation group. The mean follow up was 16 months and 39% of patients were female. Study and participant characteristics are further detailed on Table 1 .

**Table 1:** – Baseline characteristics of included studies

	NAVIGATE ESUS^11^	RE-SPECT ESUS^10^	ATTICUS^9^	ARCADIA^8^
Year of Publication	2018	2019	2023	2024
Population, n (DOAC/ASA)	7213 (3609/3604)	5390 (2695/2695)	352 (178/174)	1015 (507/508)
Age, mean (SD) (DOAC/ASA)	66.9 (9.8)/66.9 (9.8)	64.2 (11.4)/63.9 (11.4)	68.6 (10.5)/68.3 (48.9)	67.8 (10.8)/68.2 (11.0)
Female, n (%) (DOAC/aspirin)	1377 (38.15)/ 1400 (38.84)	1001(37.1)/ 986 (36.6)	86 (48.3)/ 85 (49.9)	272 (53.7)/ 279 (54.9)
BMI (SD) Kg/m^2^(DOAC/aspirin)	27.1 (4.9)/27.3 (5.1)	27.2 (5)/27.3 (5)	27.7 (5.2)/27.7 (4.9)	N/A
**Comorbidities**				
Hypertension, n (%) (DOAC/ aspirin)	2782 (77)/ 2803 (78)	1996 (74.1)/ 1985 (73.7)	153 (86)/ 150 (86.2)	396 (78.1)/ 388 (76.4)
Diabetes, n (%) (DOAC/ASA)	889 (25)/ 917 (25)	485 (21.7)/ 639 (23.7)	52 (29.2)/ 48 (27.6)	156 (30.8)/ 159 (31.3)
Prior stroke or TIA, n (%) (DOAC/ aspirin)	620 (17.5)/643 (18)	475 (17.6)/500 (18.6)	24(13.5)/30 (17.2)	97 (19.1)100 (19.7)
Ischemic or coronary artery disease, n (%) (DOAC/ aspirin)	N/A	301 (11.2)/276 (10.2)	12 (6.7)/17 (9.8)	58 (11.4)/46 (9.1)
Heart failure, n (%) (DOAC/ aspirin)	N/A	117 (4.3)/ 124 (4.6)	3 (1.7)/ 4 (2.3)	36 (7.1)/35 (6.9)
Prior* or current tobacco use, n (%) (DOAC/ aspirin A)	756 (21)/ 728 (20)	458 (17)/ 433 (16.1)	27 (15.2)/ 26 (14.9)	230 (45.4) /200 (39.4)*
Peripheral arterial disease, n (%) (DOAC/aspirin)	N/A	N/A	7 (3.9)/9 (5.2)	12 (2.4)/ 7 (1.4)
PFO, n (%) (DOAC/aspirin)	NA	319 (11.8)/361 (13.4)	44 (26.3)/24 (14.5)	N/A
CHA_2_DS_2_-VASc score, mean (SD) (DOAC/aspirin)	N/A	N/A	5 (4–6)/5 (4-5)	4.7 (1.3)/4.7 (1.3)
NIH Stroke Scale score, median (IQR) (DOAC/ aspirin)	1 (0–2)/1 (0-2)	1 (0–2)/1 (0-2)	1 (0–3)1 (0-3)	1 (0-3)/1 (0-3)
Days from index stroke to randomization, median (IQR) (DOAC/aspirin)	38.0 (15.0–89.0)/ 36 (14.0-86.5)	46.0 (21.0–82.0)/ 43.0 (20.0-78)	8 (6–12)/ 8 (6-12)	48 (21-96)/ 53 (23-100)

BMI: body mass index; DOAC: direct oral anticoagulants; IQR: interquartile range; NIH: National Institutes of Health; PFO: patent foramen ovale; TIA: transient ischemic accident; SD: standard deviation.

### Outcomes

#### Stroke-related outcomes

Compared with aspirin, DOACs did not decrease the incidence of recurrent stroke (HR: 0.95; 95% CI: 0.83-1.09; p=0.44; I^2^=0%; Figure 2A ), the composite of recurrent stroke or systemic embolism (HR: 1,03; 95% CI: 0.86-1.24; p=0.75; I^2^=0%; Figure 2B ), recurrent ischemic stroke (HR: 0.91; 95% CI: 0.79-1.06; p=0.23;I^2^=0%; Figure 2C ), and hemorrhagic stroke (HR: 2.21; 95%CI: 0.31-16.02; p=0.43; I^2^=78%; Figure 2D ). Disabling stroke was reported only by 2 studies and there were no significant differences between groups (HR: 0.91; 95% CI: 0.39-2.16; p=0.84; I^2^=84%; Supplemental Figure 1 ).

**Figure 2:** – There were no significant differences between direct oral anticoagulants (DOACs) and aspirin therapy in patients with embolic stroke of undetermined source (ESUS) regarding (A) recurrent stroke; (B) the composite outcome of recurrent stroke or systemic embolism; (C) ischemic stroke; and (D) hemorrhagic stroke.

#### Mortality outcomes

There were no significant differences between groups in the incidence of all-cause mortality (HR: 1.11; 95% CI: 0.87-1.42; p=0.40; I^2^=0%; Figure 3A ) and cardiovascular mortality (HR: 1.12; 95%CI: 0.75-1.66; p=0.58; I^2^=18%; Figure 3B ).

**Figure 3:** – There were no significant differences between direct oral anticoagulants (DOACs) and antiplatelet therapy in patients with embolic stroke of undetermined source (ESUS) regarding (A) all-cause mortality; (B) cardiovascular mortality; and (C) myocardial infarction.

#### Myocardial infarction

MI was reported by three studies. No significant differences were observed between groups (HR: 0.94; 95% CI: 0.61-1.44; p=0.77; I^2^= 17%; Figure 3C ).

#### Bleeding outcomes

There were no significant differences between groups in the incidence of major bleeding (HR: 1.56; 95%CI: 0.85-2.86; p=0.15; I^2^=64%; Figure 4A ). However, compared with aspirin, DOACs significantly increased the incidence of CRNMB (HR: 1.54; 95% CI: 1.23-1.92; p=0.0002; I^2^=8%; Figure 4B ).

**Figure 4:** – Compared with antiplatelet therapy, direct oral anticoagulants (DOACs) significantly increased the incidence of (A) clinically relevant non-major bleeding (CRNMB); there were no significant differences between groups in (B) major bleeding.

#### Subgroup analyses

There were no significant differences in stroke recurrence between DOAC and aspirin therapy in both the subgroup of patients with low CHA_2_-DS_2_-VASc score (defined as ≤4) (RR: 0.87; 95% CI: 0.67-1.13; p=0.28; I^2^=0%; Supplementary Figure 2A ) and the subgroup of patients with high CHA_2_DS_2_-VASc score (defined as ≥ 5) (RR: 0.83; 95% CI: 0.65-1.07; p=0.16; I^2^=0%; Supplementary Figure 2B ).

#### Quality assessment and sensitivity analysis

As depicted in the Supplementary Figure 3 , all included studies were deemed to have a low risk of bias.^8^The leave-one-out sensitivity analysis for the outcome of CRNMB yielded consistent results, showing no study dominance ( Supplementary Figure 4 ). The funnel plot analysis of the outcome CRNMB did not demonstrate any potential small study effect with a symmetric graphical distribution of studies with similar weights against their standard errors ( Supplementary Figure 5 ).

## Discussion

In this meta-analysis of four RCTs, DOACs were compared to aspirin in 13,970 patients with a history of ESUS. The key findings were as follows: we observed no statistically significant differences between the two groups in terms of recurrent stroke, composite outcomes of recurrent stroke or systemic embolism, ischemic stroke, hemorrhagic stroke, all-cause mortality, cardiovascular mortality, myocardial infarction, major bleeding, or disabling stroke. However, patients treated with DOACs exhibited a higher incidence of CRNMB compared to those managed with aspirin.^12^

The premise that oral anticoagulation is more effective than antiplatelet therapy in preventing strokes related to AF is well-established.^15^Given the high rate of AF detection, which may range up to 16% in patients with cryptogenic stroke during 3 to 12 months of continuous cardiac monitoring, the rationale for exploring anticoagulation in this context is compelling.^16 , 17^

Early trials comparing DOACs with aspirin demonstrated no clear superiority of anticoagulation in patients with ESUS.^10 , 11^These findings prompted further trials to investigate certain subsets of ESUS patients, including those with increased risk of AF and cardioembolism, such as individuals with atrial cardiopathy.^8 , 9^In a pivotal study^, 9^, this approach gave rise to the concept of “enriched ESUS”, as distinguished from the “unselected ESUS” population that participated in previous studies. Despite these efforts to stratify ESUS patients more precisely, subsequent trials mirrored the initial findings of no superiority of anticoagulation over antiplatelet therapy.^8 , 9^

A sub-study of the ARTESIA trial has shown that the baseline CHA_2_DS_2_-VASc score helps to identify and guide oral anticoagulation in patients with subclinical atrial fibrillation.^18^For CHA_2_DS_2_-VASc score > 4, the benefits of treatment with apixaban in preventing embolic stroke was greater than the risks. We found no significant differences between the groups regarding stroke recurrence in the subgroup of patients with low and high CHA_2_DS_2_-VASc scores.^19^

In a previous published meta-analysis of two RCTs, anticoagulation therapy neither decreased the recurrence of stroke nor escalated bleeding events, including major bleeding and CRNMB events when compared to antiplatelet therapy.^12^In alignment with these results, we found no significant difference in the efficacy of DOACs for the management of ESUS. However, our findings suggest that this therapy modality might increase the risk of CRNMB for this population.

Considering these collective findings, our meta-analysis underscores the absence of discernible benefit from anticoagulation in ESUS patients across various risk profiles. Despite the compelling rationale for anticoagulation based on AF detection rates and the theoretical pathophysiological considerations,^4 - 7^available data failed to demonstrate that therapy with DOACs is superior to aspirin in reducing the recurrence of strokes among ESUS patients.

To the best of our knowledge, this is one of the largest meta-analysis restricted to RCTs comparing DOACs with aspirin in patients with ESUS. By pooling a larger dataset, our results provide increased statistical power, offering a valuable perspective into the ongoing debate surrounding stroke management and prevention strategies. Moreover, our study has considerable strengths as rigorous assessment determined that all included trials had a low risk of bias. Most of these trials utilized a double-blind design,^8 , 10 , 11^along with blinded outcome adjudicators,^8 - 11^which significantly mitigates the potential for bias.

Although it provides valuable insights, our study has limitations. First, we observed variations between studies with regards to comorbidity definitions, trial selection criteria, and in follow-up durations. Second, the follow-up duration estimates were based on reported averages, potentially overlooking significant treatment benefits suggested by late divergence in Kaplan–Meier curves, as observed in RE-SPECT ESUS. Moreover, we observed a moderate to high heterogeneity in the major bleeding outcome, which might reflect the above-mentioned variations between studies. This was approached by using a random-effects analysis to mitigate its influence in the pooled analysis. Lastly, although a broad search was performed for studies comparing anticoagulation therapy with antiplatelet therapy, all the included studies exclusively compared DOACs to aspirin. Consequently, our post-search analysis was specifically narrowed to DOACs versus aspirin, excluding comparisons with other forms of anticoagulation or antiplatelet treatments. Thus, the outcomes of our meta-analysis may not be broadly applicable to alternative anticoagulant or antiplatelet regimens.

Atrial cardiopathy markers such as left atrial volume index (LAVI) and NT-pro-BNP levels show potential for stratifying ESUS outcomes. However, due to absence of patient-level data, this meta-analysis could not explore such subgroups. Regarding individual studies, the ARCADIA trial found no significant difference in stroke recurrence between apixaban and aspirin in patients with NT-pro-BNP >250 pg/mL or a left atrial diameter index >1.8 cm^2^.^8^However, other trials, including ATTICUS, NAVIGATE ESUS, and RE-SPECT ESUS, did not report subgroup analyses for these markers.^9 - 11^Future research integrating atrial cardiopathy markers may better identify subgroups that benefit from anticoagulation therapy.

Therefore, in selecting treatment strategies for patients with ESUS, healthcare providers must carefully weigh the potential benefits of DOACs against its associated bleeding risks. Overall, our results support that antiplatelet therapy with aspirin might be a safer option for patients with ESUS. However, it is important to mention that its use remains with a substantial risk of recurrent stroke in the ESUS population, which highlights the importance of exploring other approaches. Thus, future research is vital for identifying alternative management strategies and enhancing outcomes for ESUS patients.

## Conclusion

In this meta-analysis of four RCTs, we found that DOACs use was not superior to aspirin in preventing recurrent stroke in patients with ESUS. Moreover, there was a statistically significant increase in CRNMB events in the DOACs group as compared with the aspirin group, despite a not significant difference in the incidence of major bleeding.

## *Supplemental Materials

For additional information, please click here.
