# Supplementary material for: Errata
Source: Arq Bras Cardiol. 2025 Aug 6;122(7):e20250535. [Article in Portuguese] doi: 10.36660/abc.20250535 (PMC12680086; doi:10.36660/abc.20250535)
Supplement: Translation

# Translation: Erratum

**DOI:** 10.36660/abc.20250535i

**June 2025 Issue, vol. 122(6):e20240586**

In the Review Article "Direct Oral Anticoagulants versus Aspirin for Secondary Stroke Prevention in Patients with Embolic Stroke of Undetermined Source: An Updated Systematic Review and Meta-Analysis of Randomized Controlled Trials", with DOI: https://doi.org/10.36660/abc.20240586i , published in the journal Arquivos Brasileiros de Cardiologia, Arq Bras Cardiol. 2025; 122(6):e20240586, on page 1, change the name of the author Thomas Costa Alexandre to: Thomaz Alexandre Costa.
